# Control of Streptococcal Infections: Is a Common Vaccine Target Achievable Against *Streptococcus agalactiae* and *Streptococcus pneumoniae*

**DOI:** 10.3389/fmicb.2021.658824

**Published:** 2021-04-23

**Authors:** Edmund Bedeley, Andrea Gori, Dorothy Yeboah-Manu, Kanny Diallo

**Affiliations:** ^1^Department of Bacteriology, Noguchi Memorial Institute for Medical Research, University of Ghana, Accra, Ghana; ^2^NIHR Global Health Research Unit on Mucosal Pathogens, Division of Infection and Immunity, University College London, London, United Kingdom; ^3^West African Centre for Cell Biology of Infectious Pathogens, College of Basic and Applied Sciences, University of Ghana, Accra, Ghana; ^4^Centre Suisse de Recherche Scientifique de Côte d’Ivoire, Abidjan, Côte d’Ivoire

**Keywords:** *Streptococcus agalactiae*, *Streptococcus pneumoniae*, genome sequence, target, vaccine

## Abstract

Both *Streptococcus agalactiae* [group B streptococcus (GBS)] and *Streptococcus pneumoniae* (pneumococcus) remain significant pathogens as they cause life threatening infections mostly in children and the elderly. The control of diseases caused by these pathogens is dependent on antibiotics use and appropriate vaccination. The introduction of the pneumococcal conjugate vaccines (PCVs) against some serotypes has led to reduction in pneumococcal infections, however, the subsequent serotype switching, and replacement has been a serious challenge. On the other hand, no vaccine is yet licensed for use in the control of GBS diseases. In this review, we provide an overview of the history and global disease burden, disease pathophysiology and management, vaccines update, and the biology of both pathogens. Furthermore, we address recent findings regarding structural similarities that could be explored for vaccine targets across both mucosal pathogens. Finally, we conclude by proposing future genomic sequence comparison using the wealth of available sequences from both species and the possibility of identifying more related structural components that could be exploited for pan-pathogen vaccine development.

## Introduction

*Streptococcus agalactiae* (Lancefield group B streptococcus; *S. agalactiae*, GBS) and *Streptococcus pneumoniae* (*S. pneumoniae*; pneumococcus) are two important mucosal pathogens responsible for the leading cause of invasive disease in newborns, pregnant women, and the elderly, with occasional incidence in healthy adults (Le Doare and Heath, 2013). Both pathogens are implicated in pneumonia, sepsis, and meningitis (Salloum et al., 2011; Torné et al., 2014). Asymptomatic carriage is a prerequisite of invasive infection caused by these organisms (Gray et al., 1980). Pneumococcal conjugate vaccines (PCVs) and pneumococcal polysaccharide vaccines (PPVs) are available against some serotypes; PCVs are effective in reducing invasive disease caused by vaccine serotypes (Berical et al., 2016). No vaccine has been licensed yet for GBS as clinical trials are still ongoing (Lin et al., 2018). Several other challenges such as serotype replacement in *S. pneumoniae* and ineffectiveness of GBS vaccines (in clinical trial) in late onset disease (LOD) remain significant (Hsu et al., 2010; Thigpen et al., 2011; Berical et al., 2016).

In the past 20 years, few published studies suggested some possible useful similarities observed within the structural genes and protein sequences between GBS and *S. pneumoniae* (Guttormsen et al., 2002; Jarva et al., 2004; Löfling et al., 2011). This review highlights commonalities across both streptococcal species and explores the possibility of identifying common vaccine targets for both pathogens.

## Materials and Methods

The literature review focused on the history, disease burden, disease pathophysiology and management, vaccines update, and findings regarding structural similarities that could be explored for vaccine targets across both mucosal pathogens. Three main literature search strategies were used with the advanced search option of the PUBMED database. The first one was to obtain literature on both GBS and *S. pneumoniae* together using the search terms ((((((group b streptococcal[Title/Abstract]) OR (group b streptococcus[Title/Abstract])) OR (*Streptococcus agalactiae*[Title/Abstract])) AND (*Streptococcus pneumoniae*[Title/Abstract])) OR (pneumococcus[Title/Abstract])) OR (pneumococcal[Title/Abstract]) using the title/abstract option in the query box. This search yielded 18,207 papers. The second and third criteria were to obtain literature on GBS and *S. pneumoniae* separately using the search terms (((group b streptococcal[Title/Abstract]) OR (group b streptococcus[Title/Abstract])) OR (*Streptococcus agalactiae*[Title/Abstract])) AND (disease[Title/Abstract]) and (((*Streptococcus pneumoniae*[Title/Abstract]) OR (pneumococcus[Title/Abstract])) OR (pneumococcal[Title/Abstract])) AND (disease[Title/Abstract]), respectively, which yielded a total of 9,415. In all, 79 relevant studies including some reference listed in the identified articles were screened through their titles and abstracts and included in this study. The literature search was conducted in July 2019 and updated in March 2021.

## Historical Overview and Epidemiology of GBS and *S. Pneumoniae* Infections

Group B streptococcus was first isolated in bovine mastitis (Nocard and Mollereau, 1887) and subsequently from vaginal swabs (Lancefield and Hare, 1935). Fry described three fatal incidences of GBS infections in post-partum women, but before this finding, most severe streptococcal infections implicated group A streptococcus (GAS; Fry, 1938). In the 1960s, a number of GBS neonatal sepsis were recognized in many developed countries especially, the United States and the United Kingdom (Hood et al., 1961; Eickhoff et al., 1964; Kalliola et al., 1999; Fluegge et al., 2006; Neto, 2008).

The WHO identified Africa as the most affected region, with the highest GBS invasive infection in infants (0–89 days; Edmond et al., 2012) and similarly in 2017, Madrid et al. (2017) estimated more than double infant GBS disease in Africa compared to developed countries. In a global report, the lowest disease incidence was estimated in Southeast Asia, with the worst affected areas being low/middle income countries (LMIC) which face many challenges ranging from lack of baseline information on GBS infection to failure to collect samples in areas with high GBS neonatal mortality (Edmond et al., 2012; Sinha et al., 2016; Kobayashi et al., 2019). High-income countries (HIC) such as the United States and the United Kingdom still report close to 2,000 incidences of infant invasive GBS infection (Kobayashi et al., 2019), and GBS associated stillbirths every year (Turrentine and Ramirez, 2008; Seale et al., 2017).

*Streptococcus pneumoniae* was first identified by Pasteur and Sternberg independently from saliva in 1881 (White, 1938). Friedlander and Talamon first associated lobular pneumonia to the pathogen in 1883. Subsequently (between 1915 and 1945), extensive studies were conducted to better understand the medical importance of the capsular polysaccharide of *S. pneumoniae*, its virulence and antigenicity (CDC, 2021). *S. pneumoniae* is the most common pathogen implicated in community-acquired pneumonia (CAP), accounting for about 25–30% of cases (Welte et al., 2012). A high mortality rate was reported in pneumococcal global disease burden (Berkelman et al., 2006; Klugman et al., 2008), with over 1 million deaths recorded annually – the worst affected age group being children under 5 years old (Obaro et al., 1996). Also, out of 8.8 million deaths recorded worldwide among children under 5 years in a Kim et al. (2016) report, pneumococcal infections were responsible for approximately half a million deaths (Kim et al., 2016). However, following the introduction of the PCVs, a decline in pneumococcal diseases has been noted (Megiddo et al., 2018).

## Serotypes of GBS and *S. Pneumoniae*

There are 10 serotypes (Ia, Ib, II, III, IV, V, VI, VII, VIII, and IX) of GBS defined based on the capsular polysaccharide (Lancefield, 1934). A global review of invasive isolates showed that serotype III (48.9%) was the most commonly identified across all regions; and this was followed by serotypes Ia (22.9%), V (9.1%), Ib (7.0%), and II (6.2%; Edmond et al., 2012; Le Doare and Heath, 2013). In a more recent study, GBS serotypes I–V accounted for 98% in colonization with serotype III associated with 25% invasive disease in most parts except Asia and South America. In Asia serotype, VI–IX were more common (Russell et al., 2017).

At least 100 different *S. pneumoniae* serotypes have been identified and all serotypes are suspected to cause serious human infections (Geno et al., 2015; Ganaie et al., 2020). For instance, in 1992, 77 out of 84 serotypes were identified in invasive disease (Nielsen and Henrichsen, 1992). Even though the introduction of pneumococcal vaccines saw an increase in level of protection, especially in children, several studies now report non-vaccine types (NVTs) in invasive pneumococcal disease (IPD; Balsells et al., 2018); this also confirm the ability of most capsular serotypes to cause disease. However, it is worth noting that differences in virulence were observed based on the type of capsular polysaccharide (Brueggemann and Peto, 2004; Sandgren et al., 2004, 2005).

## Disease Pathophysiology of GBS and *S. Pneumoniae*

Group B streptococcus is a commensal organism of the lower gastrointestinal and vaginal flora of about 25–40% of healthy adult women (Edwards et al., 1985). Occasionally, GBS moves across the epithelial cells into the bloodstream to cause invasive disease (Edwards et al., 2016). Disease caused by GBS is divided into two major distinctive clinical manifestations: early onset disease (EOD) or late onset disease (LOD; Schrag et al., 2000). EOD is responsible for more than 65% of GBS infection and takes place within the 1st week of birth. EOD manifests itself with pneumonia or sepsis. Primary source of early onset (EO) infection occurs by acquisition from gastrointestinal and/or vaginal tracts, as well as from mother-to-child during childbirth (vertical transmission). LOD GBS infection on the other hand is contracted from community sources, perinatally and nosocomially; meningitis accounts for about 50% LOD (Bergeron et al., 2000; Weisner et al., 2004).

The pneumococcus is a commensal of the oropharynx (Neto et al., 2003). *S. pneumoniae* disease can be described as a pandemic disease, endemic across the world (Kalin, 1998). The major forms of disease clinical presentations are otitis media, pneumonia, bacteraemia, and meningitis (Henriques-Normark and Tuomanen, 2013; CDC, 2021). Pneumonia is the highest recorded with 5–7% case fatality. Complications such as empyema and lung abscess formation may occur. *S. pneumoniae* is identified as one of the bacterial pathogens causing meningitis, especially in countries within the African meningitis belt. Outbreaks were reported recently: for instance, in the Brong-Ahafo region of Ghana in 2016 (Kwambana-Adams et al., 2016) and across Northern parts of Ghana in April 2020 (Mensah et al., 2020).

## Disease Management in GBS and *S. Pneumoniae*

In 2015, the WHO recommended the use of intra-partum antibiotic prophylaxis (IAP) for pregnant women who had been colonized with GBS in order to help prevent vertical transmission in the early developmental stages of neonates (WHO, 2015). IAP is also administered to pregnant women in preterm pre-labor in case of amniotic membranes rupture or disruption; however, it is not recommended for pregnant women who have intact amniotic membranes, or for those with pre-labor rupture of membranes at term or near term (36 weeks gestation and above; WHO, 2015).

For *S. pneumoniae*, antibiotic resistance has been reported from isolates across the globe. It is, therefore, recommended that treatment includes a broad-spectrum cephalosporin, and often vancomycin is used for pneumococcal infections until results from antibiotic sensitivity testing are available. It is interesting to note that some antibiotics like penicillin are regaining efficacy in treatment (CDC, 2021) Pneumococcal vaccines also played a significant role in the reduction of pneumococcal disease burden. Over the years, several vaccines were approved for the prevention of pneumococcal infections but instances of serotype replacement represent major global hurdles (Berical et al., 2016).

## Overview of GBS and *S. Pneumoniae* Genome

Whole genome sequencing of GBS serogroup III strain NEM316 isolated from a fatal case of septicaemia allowed the characterization of GBS genome as a circular chromosome of 2,211,485 base pairs (bp) with a G+C content of 35.6% (EMBL accession number AL732656, Glaser et al., 2002). This is lower than those of related species such as *S. pyogenes* (38.5%; Ferretti et al., 2001) and *S. pneumoniae* (39.7%; Tettelin et al., 2001), but similar to the G+C content of *Lactococcus lactis* (*L. lactis*; 35.4%), which is a distantly related species (Bolotin et al., 2001). Virulence in GBS has been associated with the extracellular components such as the surface proteins, capsular polysaccharide, and secreted proteins (Glaser et al., 2002).

The completely annotated genome of the *S. pneumoniae* serotype 4 (TIGR4) clinical isolate encoding 2,236 predicted proteins consists of a single circular chromosome of 2,160,837 base pairs (2.16 Mbp) with a G+C content of 39.7% (Gen-Bank accession number AE005672; Tettelin et al., 2001; Henriques-Normark and Tuomanen, 2013). Variation in the genome of *S. pneumoniae* is based on its natural competence, which allows the bacterium to acquire exogenous DNA; insertion sequences (ISs) and truncated genes account for about 5% of the genome (Tettelin et al., 2001; Henriques-Normark and Tuomanen, 2013). Next generation sequencing technique has allowed to determine that the pneumococcus undergoes gradual recombination within the nasopharynx which may suggest *in vivo* DNA transfer (Hiller et al., 2010).

## The State of GBS and *S. Pneumoniae* Vaccines

Various formulations of GBS vaccines are being tested in clinical trials, but none has been approved at the time of writing (Lin et al., 2018; Table 1). These include the Novartis trivalent conjugate vaccine ((NCT01193920; Palmeiro et al., 2011; Lin et al., 2018); the pentavalent GBS PCV (Ia, Ib, II, III, and V; NCT03170609) by Pfizer (Lin et al., 2018); and the formulation of GBS pilus (Margarit et al., 2009; Nuccitelli et al., 2015). A recent study reported four candidate biomarkers (thioredoxin, CsbD-like protein, RpL7/L12, and exoDNase) that may be considered for further studies on GBS pathophysiology and for the development of novel vaccines (Lanotte et al., 2013; Nagao, 2015). A previous study showed a systemic and mucosal immune response activity by the encapsulating C5a peptidase in mice (Santillan et al., 2011). However, there are some challenges with progresses made in the GBS vaccine development: some have poor immunogenicity, some of the GBS conjugate vaccines also interfere with other conjugate vaccines like those against pneumococcal, meningococcal and the influenza type b (Chen et al., 2013; Leroux-Roels et al., 2016). The rising issues of serotype switching and replacement are another potential limitation (Teatero et al., 2017; Lin et al., 2018). Furthermore, the increase in non-encapsulated GBS strains causing diseases, calls for the evaluation of other targets as vaccine candidate (Flores et al., 2015; Teatero et al., 2017). Unlike the pneumococcal vaccines, where ELISA and multiplex-opsonohagocytosis assay (MOPA) are acceptable standards for measuring CPS-specific antibody and functional antibody titers, the gold standard for measuring antibody titers for GBS, the radio-antigen binding assay (RABA) is limited in sensitivity and unable to quantify Ig isotypes (Lin et al., 2018) making the evaluation of vaccines even more difficult.

**Table 1:** Summary of different group B streptococcus (GBS) vaccine candidates.

GBS vaccine candidates	Type	Status	Reference
Trivalent conjugate vaccine (Ia, Ib, and III; NCT01193920)	Conjugate	In clinical trial	Palmeiro et al., 2011; Lin et al., 2018
Pentavalent GBS PCV (Ia, Ib, II, III, and V; NCT03170609)	Conjugate	In clinical trial	Lin et al., 2018
GBS pilus formulation	Protein	Preclinical trial	Gianfaldoni et al., 2007; Margarit et al., 2009
Candidate biomarkers (thioredoxin, CsbD-like protein, RpL7/L12 and exoDNase)	Protein	Preclinical trial	Lanotte et al., 2013; Nagao, 2015
C5a peptidase	Protein	Preclinical trial	Santillan et al., 2011

Pneumococcal vaccines have been in existence for over a century; the first being the whole-cell vaccine in 1911 (Wright et al., 1914). The 23-valent pneumococcal polysaccharide vaccine (PPV23) was developed to replace the 14-valent vaccine (PPV14; Robbins et al., 1983). However, the use of PPV23 was associated with limitations such as: short memory development in immune cells, lack of carriage prevention in most populations, and poor ability to induce immunity in children aged less than 2 years (Centers for Disease Control and Prevention, 2010; Russell et al., 2010). Earlier studies suggested that conjugated vaccines were more immunogenic compared to unconjugated vaccines (Avery and Goebel, 1931). Therefore, PCV-7 was developed and approved in 2000 for use based on pneumococcal serotypes commonly found in invasive disease in the United States (Hsu et al., 2010; Berical et al., 2016). Varying protective effects were noted in different populations (Elston et al., 2012). Subsequently, PCV-10 vaccine was introduced and included serotypes 1, 5, and 7F which were of clinical relevance in other areas of the word such as Asia and Africa. With the increasing number of serotype-replacement and antibiotic-resistant clones observed after the introduction of PCV-10, PCV-13 was developed to include serotypes 3, 6B, and 19A. Currently, PCV-15 and PCV-20 vaccines efficacies are been evaluated in phase III trials (Pichichero, 2017). Efforts are been made for pneumococcal protein vaccine development, which will help overcome some challenges associated with the use of PCVs. These include lack of coverage against all serotypes implicated in disease, cost involved in PCV production, and the complexity of the manufacturing process. Thus, additional protein vaccine candidates are under consideration. Notable among them are pneumococcal surface protein A (PspA), PhtD, StkP, and pneumolysin (Ginsburg et al., 2012; Lagousi et al., 2019; Table 2).

**Table 2:** Summary of different pneumococcal vaccine candidates.

Pneumococcal vaccine candidates	Type	Status	Reference
23-valent pneumococcal polysaccharide vaccine (PPV23)	Polysaccharide	In use	Robbins et al., 1983
Pneumococcal conjugate vaccines (PCV-7, PCV-10, and PCV-13)	Conjugate	In use	Hsu et al., 2010; Berical et al., 2016
Pneumococcal conjugate vaccines (PCV-15 and PCV-20)	Conjugate	In clinical trial	Pichichero, 2017
Pneumococcal protein vaccines (PspA, PhtD, StkP, and pneumolysin)	Protein	In clinical trial	Ginsburg et al., 2012; Lagousi et al., 2019

PspA, pneumococcal surface protein A; PhtD, pneumococcal histidine triad protein D; StkP, serine/threonine kinase protein.

## Structural and Functional Similarities Between GBS and *S. Pneumoniae* and Opportunity for Common Vaccine Development

Guttormsen et al., in 2000 provided the first data showing that antibody response elicited by either unconjugated type III GBS polysaccharide (IIIPS), or conjugated type III GBS polysaccharide covalently linked to tetanus toxoid vaccine (III-TT) are opsonic for both GBS III and *S. pneumoniae* type 14 (Pn14). In this *in vitro* study, the immune response recruited by the GBS vaccine (IIIPS and III-TT) cross-reacted with Pn14 and killed it. This suggests that the use of GBS III vaccine could confer protection against the two pathogens implicated in invasive disease (Guttormsen et al., 2002). However, the reverse of this study did not produce similar results. Previously, another study found that despite the structural similarity between GBS IIIPS and Pn14, specific antibodies induced by unconjugated Pn14 vaccine was not opsonic for GBS III and could not kill it (Kasper et al., 1979; Baker et al., 1980). These findings suggest that it may be possible for a vaccine target in one pathogen to serve a good vaccine candidate for another pathogen.

**Figure 1:**
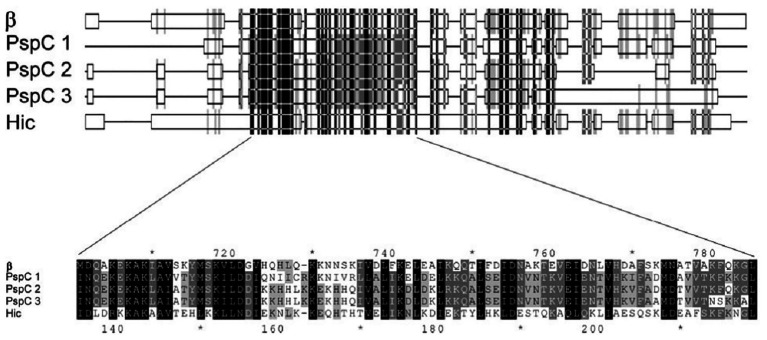
Part of the alignment of the amino acid sequences of β, three pneumococcal surface protein C (PspC) proteins, and Hic (Adapted from Jarva et al., 2004).

In 2004, Jarva et al. demonstrated that the structural homology observed between the amino acid sequences of GBS β protein and pneumococcal Hic protein, enable these pathogens to inhibit complement deposition in similar fashion (Figure 1). They employed Basic Local Alignment Search Tool (BLAST) analysis to identify similarities to the pneumococcal Hic protein and found closest similarities with the GBS β protein and several pneumococcal surface protein C (PspC). The amino acid sequences of three of the PspC proteins (GenBank accession no. AAF73789.1, AAD31043.1, and AAF73802.1) were aligned to Hic (AAG16729.1) and the β protein (P27951) of GBS, revealing that the GBS β protein is more closely related to pneumococcal PspC than to the M proteins of group A streptococci (GAS). Both β and Hic proteins were evaluated for their binding capacities for factor H (fH). This study identified multiple binding sites (SCR8–11 and SCR12–14) between β and Hic proteins by which they obtain high avidity binding to fH and obstruct opsonization (Jarva et al., 2004). *In vivo* mouse model studies showed that PspC/CbpA posed high vaccine efficacy among other non-serotype dependent pneumococcal surface proteins (Briles et al., 2000). Similarly, the beta protein of GBS was shown to elicit protective immunity in mouse models (Michel et al., 1991; Persson et al., 2008). Therefore, the similarity observed between the beta and PspC and their functional properties are of cardinal importance for pan-pathogen vaccine investigation.

**Figure 2:**
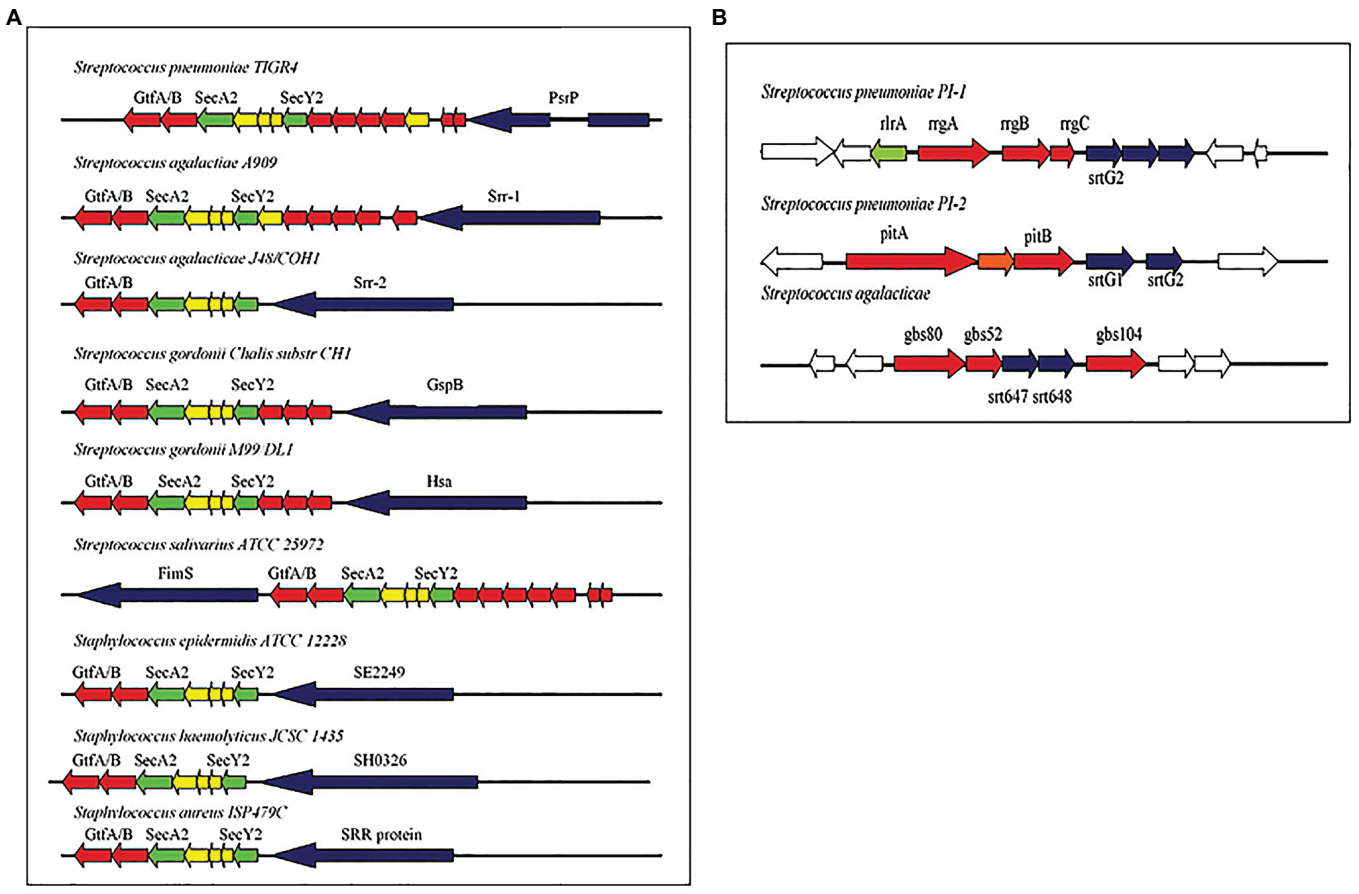
**(A)** Pathogenicity islets encoding serine-rich repeat (SRR) proteins in different streptococcal species. **(B)** Pathogenicity islets encoding pili in *Streptococcus pneumoniae* (PI-1 and PI-2) and in *Streptococcus agalactiae* (Adapted from Löfling et al., 2011).

Over the years, similarities have also been established in the cell wall adhesins of *S. pneumoniae* and other Streptococci including GBS (Giefing et al., 2008; Moriel et al., 2008). In a 2011 review of cellular interaction by pneumococcal adhesins and their streptococcal homologues, Löfling et al. (2011) discussed pili and serine rich repeat (SRR) proteins in several streptococcus species (Figure 2). The implication of this is that there could be similar path to pathogenesis and hence immune defense response mechanisms. Indeed, *in vivo* studies with mice have shown that *S. pneumoniae* and GBS pili could serve as protective antigens hence could be used in multi-component vaccines (Gianfaldoni et al., 2007; Löfling et al., 2011). Observed similarities identified in the surface structural components could be beneficial in developing a more general Streptococci vaccine. Indeed an antigen with immunological potential conserved in different streptococcal species would serve as a good vaccine candidate for a pan-species vaccine. This approach will require studies that first identify novel conserved surface proteins in multiple species. Although, this review focuses on the two streptococcal species involved in meningitis worldwide, studying similarities can be extended to other species and indeed even to other bacteria. However, it is important to keep in mind that the developement and implementation of such vaccines will need to be accompanied by monitoring of its effect on the normal flora and in this particular example, the other viridian streptococci.

## Conclusion

Group B streptococcus and *S. pneumoniae* remain important cause of neonatal diseases. The variabilities within the capsular polysaccharide of the numerous serotypes of *S. pneumoniae* pose challenges to the available PCVs-warranting more inclusion of other serotypes in new PCVs under development. No vaccine is licensed for prevention of GBS disease. Based on the observed similarities between GBS and *S. pneumoniae* and the increased number of sequenced GBS and *S. pneumoniae* isolates, it would be prudent to perform genomic comparison across the various serotypes of *S. pneumoniae* and GBS using the available genomic analysis tools in order to revisit those hypotheses that common structural components among both species could elicit a cross reactive immunity. This would allow a better understanding of the already defined immune cross interactions between both species and potentially identify new ones. One approach would be to define a common core genome between these two species of Streptococci and study structural genes with high level of similarity across their serotypes in order to identify immunogenic proteins that are homologous and that could be used as novel vaccine candidates antigen capable of targeting both species at once. Identifying new common structural components will shed a light on the possibility of developing and implementing a pan-pathogen vaccine, conferring a protection against multiple mucosal pathogens.

## Author Contributions

KD and AG developed the idea, provided the support, and made substantive impute into the manuscript. EB wrote the manuscript. DY-M provided the supervision and made intellectual impute into the manuscript. All authors contributed to the article and approved the submitted version.

### Conflict of Interest

The authors declare that the research was conducted in the absence of any commercial or financial relationships that could be construed as a potential conflict of interest.

## References

[ref2] Avery O. Goebel W. (1931). Chemo-immunological studies on carbohydrate-proteins. V. The immunological specificity of an antigen prepared by combining the capsular polysaccharide of type III pneumococcus with foreign protein. J. Exp. Med. 54, 419–426. 10.1084/jem.54.3.437 PMC2132011 19869930

[ref3] Baker C. Kasper D. Edwards M. Schiffman G. (1980). Influence of preimmunization antibody levels on the specificity of the immune response to related polysaccharide antigens. N. Engl. J. Med. 303, 173–178. 10.1056/NEJM198007243030401, PMID: 6155612

[ref4] Balsells E. Dagan R. Yildirim I. Gounder P. Steens A. Muñoz-Almagro C.. (2018). The relative invasive disease potential of *Streptococcus pneumoniae* among children after PCV introduction: a systematic review and meta-analysis. J. Inf. Secur. 77, 368–378. 10.1016/j.jinf.2018.06.004, PMID: 29964140

[ref5] Bergeron M. Ke D. Menard C. Picard F. Gagnon M. Bernier M.. (2000). Rapid detection of group B streptococci in pregnant women at delivery. N. Engl. J. Med. 343, 175–179. 10.1056/NEJM200007203430303, PMID: 10900276

[ref6] Berical A. C. Harris D. Dela Cruz C. S. Possick J. D. (2016). Pneumococcal vaccination strategies. An update and perspective. Ann. Am. Thorac. Soc. 13, 933–944. 10.1513/AnnalsATS.201511-778FR, PMID: 27088424 PMC5461988

[ref7] Berkelman R. Cassell G. Specter S. Hamburg M. Klugman K. (2006). The “Achilles heel” of global efforts to combat infectious diseases. Clin. Infect. Dis. 42, 1503–1504. 10.1086/504494, PMID: 16619171

[ref8] Bolotin A. Wincker P. Mauger S. Jaillon O. Malarme K. Weissenbach J.. (2001). The complete genome sequence of the lactic acid bacterium Lactococcus lactis ssp. lactis IL1403. Genome Res. 11, 731–753. 10.1101/gr.GR-1697R, PMID: 11337471 PMC311110

[ref9] Briles D. Hollingshead S. Brooks-Walter A. Nabors G. Ferguson L. Schilling M.. (2000). The potential to use PspA and other pneumococcal proteins to elicit protection against pneumococcal infection. Vaccine 18, 1707–1711. 10.1016/S0264-410X(99)00511-3, PMID: 10689153

[ref10] Brueggemann A. Peto T. (2004). Temporal and geographic stability ofthe serogroup-specific invasive disease potential of *Streptococcus pneumoniae* in children. Infect. Dis. Ther. 190, 1203–1211. 10.1086/423820, PMID: 15346329

[ref11] CDC (2021). Epidemiology of Vaccine Preventable Diseases. Available at: https://www.cdc.gov/vaccines/pubs/pinkbook/pneumo.html (Accessed March 22, 2021).

[ref12] Centers for Disease Control and Prevention (2010). Licensure of a 13-valent pneumococcal conjugate vaccine (PCV13) and recommendations for use among children—advisory committee on immunization practices (ACIP). MMWR Morb. Mortal. Wkly Rep. 59, 258–261. PMID: 20224542

[ref13] Chen V. Avci F. Kasper D. (2013). A maternal vaccine against group B streptococcus: past, present, and future. Vaccine 31(Suppl. 4), D13–D19. 10.1016/j.vaccine.2012.12.080, PMID: 23973342 PMC3757342

[ref14] Edmond K. Kortsalioudaki C. Scott S. Schrag S. Zaidi A. Cousens S.. (2012). Group B streptococcal disease in infants aged younger than 3 months: systematic review and meta-analysis. Lancet 379, 547–556. 10.1016/S0140-6736(11)61651-6, PMID: 22226047

[ref15] Edwards M. Nizet V. Baker C. (2016). “Group B Streptococcal Infections” in Remington and Klein’s Infectious Diseases of the Fetus and Newborn Infant. eds. Wilson C. B. Nizet V. Maldonado Y. A. Remington J. S. Klein J. O. (Philadelphia: PA. Elsevier Saunders), 411–456.

[ref16] Edwards M. Rench M. Haffar A. Murphy M. Desmond M. Baker C. (1985). Long-term sequelae of group B streptococcal meningitis in infants. J. Pediatr. 106, 717–722. 10.1016/S0022-3476(85)80342-5 3889248

[ref17] Eickhoff T. Klein J. Daly A. Ingall D. Finland M. (1964). Neonatal sepsis and other infections due to group B beta-hemolytic streptococci. N. Engl. J. Med. 271, 1221–1228. 10.1056/NEJM196412102712401, PMID: 14234266

[ref18] Elston J. W. T. Santaniello-Newton A. Meigh J. A. Harmer D. Allgar V. Allison T.. (2012). Increasing incidence of invasive pneumococcal disease and pneumonia despite improved vaccination uptake: surveillance in Hull and East Yorkshire, UK, 2002-2009. Epidemiol. Infect. 140, 1252–1266. 10.1017/S0950268811001907, PMID: 22040368

[ref19] Ferretti J. McShan W. Ajdic D. Savic D. Savic G. Lyon K.. (2001). Complete genome sequence of an M1 strain of *Streptococcus pyogenes*. Proc. Natl. Acad. Sci. U. S. A. 98, 4658–4663. 10.1073/pnas.071559398, PMID: 11296296 PMC31890

[ref20] Flores A. Galloway-Pena J. Sahasrabhojane P. Al E. (2015). Sequence type 1 group B streptococcus, an emerging cause of invasive disease in adults, evolves by small genetic changes. Proc. Natl. Acad. Sci. U. S. A. 112, 6431–6436. 10.1073/pnas.1504725112, PMID: 25941374 PMC4443349

[ref21] Fluegge K. Siedler A. Heinrich B. Schulte-Moenting J. Moennig M. Bartels D.. (2006). Incidence and clinical presentation of invasive neonatal group B strepto-coccal infections in Germany. Pediatrics 117, 1139–1145. 10.1542/peds.2005-2481, PMID: 16682492

[ref22] Fry R. (1938). Fatal infections by hemolytic streptococcus group B. Lancet 1, 199–201.

[ref23] Ganaie F. Saad J. S. McGee L. van Tonder A. J. Bentley S. D. Lo S. W.. (2020). A new pneumococcal capsule type, 10D, is the 100th serotype and has a large cps fragment from an oral streptococcus. MBio 11:e00937–20. 10.1128/mBio.00937-20, PMID: 32430472 PMC7240158

[ref24] Geno K. A. Gilbert G. L. Song J. Y. Skovsted I. C. Klugman K. P. Jones C.. (2015). Pneumococcal capsules and their types: past, present, and future. Clin. Microbiol. Rev. 28, 871–899. 10.1128/CMR.00024-15, PMID: 26085553 PMC4475641

[ref25] Gianfaldoni C. Censini S. Hilleringmann M. Moschioni M. Facciotti C. Pansegrau W.. (2007). *Streptococcus pneumoniae* pilus subunits protect mice against lethal challenge. Infect. Immun. 75, 1059–1062. 10.1128/IAI.01400-06, PMID: 17145945 PMC1828493

[ref26] Giefing C. Meinke A. Hanner M. Henics T. Bui M. Gelbmann D.. (2008). Discovery of a novel class of highly conserved vaccine antigens using genomic scale antigenic fingerprinting of pneumococcus with human antibodies. J. Exp. Med. 205, 117–131. 10.1084/jem.20071168, PMID: 18166586 PMC2234372

[ref27] Ginsburg A. S. Nahm M. H. Khambaty F. M. Alderson M. R. (2012). Issues and challenges in the development of pneumococcal protein vaccines. Expert Rev. Vaccines 11, 279–285. 10.1586/erv.12.5, PMID: 22380821 PMC3777823

[ref28] Glaser P. Rusniok C. Buchrieser C. Chevalier F. Frangeul L. Msadek T.. (2002). Genome sequence of *Streptococcus agalactiae*, a pathogen causing invasive neonatal disease. Mol. Microbiol. 45, 1499–1513. 10.1046/j.1365-2958.2002.03126.x, PMID: 12354221

[ref29] Gray B. M. Converse G. M. Dillon H. C. Jr. (1980). Epidemiologic studies of *Streptococcus pneumoniae* in infants: acquisition, carriage, and infection during the first 24 months of life. J. Infect. Dis. 142, 923–933. 10.1093/infdis/142.6.923, PMID: 7462701

[ref30] Guttormsen H. Baker C. J. Nahm M. H. Paoletti L. C. Zughaier S. M. Edwards M. S.. (2002). Type III Group B streptococcal polysaccharide induces antibodies that cross-react with *Streptococcus pneumoniae* type 14. Infect. Immun. 70, 1724–1738. 10.1128/IAI.70.4.1724-1738.2002, PMID: 11895934 PMC127872

[ref31] Henriques-Normark B. Tuomanen E. I. (2013). The pneumococcus: epidemiology, microbiology, and pathogenesis. Cold Spring Harb. Perspect. Med. 3:a010215. 10.1101/cshperspect.a010215, PMID: 23818515 PMC3685878

[ref32] Hiller N. Powell E. Martin D. Eutsey R. Earl J. Janto B.. (2010). Generation of genic diversity among *Streptococcus pneumoniae* strains via horizontal gene transfer during a chronic polyclonal pediatric infection. PLoS Pathog. 6:e1001108. 10.1371/journal.ppat.1001108, PMID: 20862314 PMC2940740

[ref33] Hood M. Janney A. Dameron G. (1961). Beta hemolytic streptococcus group B associated with problems of the perinatal period. Am. J. Obstet. Gynecol. 82, 809–818. 13908742 10.1016/s0002-9378(16)36146-4

[ref34] Hsu K. Shea K. Stevenson A. Pelton S. Massachusetts Department of Public Health (2010). Changing serotypes causing childhood invasive pneumococcal disease: Massachusetts, 2001-2007. Pediatr. Infect. Dis. J. 29, 289–293. 10.1097/INF.0b013e3181c15471, PMID: 19935447

[ref35] Jarva H. Hellwage J. Jokiranta T. S. Lehtinen M. J. Zipfel P. F. Meri S. (2004). The group B streptococcal beta and pneumococcal Hic proteins are structurally related immune evasion molecules that bind the complement inhibitor factor H in an analogous fashion. J. Immunol. 172, 3111–3118. 10.4049/jimmunol.172.5.3111, PMID: 14978117

[ref36] Kalin M. (1998). Pneumococcal serotypes and their clinical relevance. Thorax 53, 159–162. 10.1136/thx.53.3.159, PMID: 9659348 PMC1745174

[ref37] Kalliola S. Vuopio-Varkila J. Takala A. Eskola J. (1999). Neonatal group *B streptococcal* disease in Finland: a ten-year nationwide study. Pediatr. Infect. Dis. J. 18, 806–810. 10.1097/00006454-199909000-00012, PMID: 10493342

[ref38] Kasper D. Baker C. Baltimore R. Crabb J. Schiffman G. Jennings H. (1979). Immunodeterminant specificity of human immunity to type III group B streptococcus. J. Exp. Med. 149, 327–339. 10.1084/jem.149.2.327, PMID: 84042 PMC2184812

[ref84] Kim Y. K. LaFon D. Nahm M. H. (2016). Indirect effects of pneumococcal conjugate vaccines in national immunization programs for children on adult pneumococcal disease. Infect Chemother. 48, 257-266. 10.3947/ic.2016.48.4.257, PMID: 28032483 PMC5204004

[ref39] Klugman K. Madhi S. Albrich W. (2008). Novel approaches to the identification of *Streptococcus pneumoniae* as the cause of community-acquired pneu-monia. Clin. Infect. Dis. 47, S202–S206. 10.1086/591405, PMID: 18986290

[ref40] Kobayashi M. Schrag S. J. Alderson M. R. Madhi S. A. Baker C. J. Sobanjo-Ter Meulen A.. (2019). WHO consultation on group B Streptococcus vaccine development: report from a meeting held on 27-28 April 2016. Vaccine 37, 7307–7314. 10.1016/j.vaccine.2016.12.029, PMID: 28017431 PMC6892266

[ref41] Kwambana-Adams B. A. Asiedu-Bekoe F. Sarkodie B. Afreh O. K. Kuma G. K. Owusu-Okyere G.. (2016). An outbreak of pneumococcal meningitis among older children (≥5 years) and adults after the implementation of an infant vaccination programme with the 13-valent pneumococcal conjugate vaccine in Ghana. BMC Infect. Dis. 16:575. 10.1186/s12879-016-1914-3, PMID: 27756235 PMC5070171

[ref42] Lagousi T. Basdeki P. Routsias J. Spoulou V. (2019). Novel protein-based pneumococcal vaccines: assessing the use of distinct protein fragments instead of full-length proteins as vaccine antigens. Vaccine 7:9. 10.3390/vaccines7010009, PMID: 30669439 PMC6466302

[ref43] Lancefield R. (1934). A serological differentiation of specific types of bovine hemolytic streptococci (group B). J. Exp. Med. 59, 441–458. 10.1084/jem.59.4.441, PMID: 19870257 PMC2132330

[ref44] Lancefield R. Hare R. (1935). The serological differentiation of pathogenic and non-pathogenic strains of hemolytic streptococci from parturient women. J. Exp. Med. 61, 335–349. 10.1084/jem.61.3.335, PMID: 19870362 PMC2133228

[ref45] Lanotte P. Perivier M. Haguenoer E. Mereghetti L. Burucoa C. Claverol S.. (2013). Proteomic biomarkers associated with *Streptococcus agalactiae* invasive genogroups. PLoS One 8:e54393. 10.1371/journal.pone.0054393, PMID: 23372719 PMC3553121

[ref46] Le Doare K. Heath P. T. (2013). An overview of global GBS epidemiology. Vaccine 31, D7–D12. 10.1016/j.vaccine.2013.01.009, PMID: 23973349

[ref47] Leroux-Roels G. Maes C. Willekens J. Al E. (2016). A randomized, observer-blind Phase Ib study to identify formulations and vaccine schedules of a trivalent group B streptococcus vaccine for use in non-pregnant and pregnant women. Vaccine 34, 1786–1791. 10.1016/j.vaccine.2016.02.044, PMID: 26928074

[ref48] Lin S. M. Zhi Y. Ahn K. B. Lim S. Seo H. S. (2018). Status of group B streptococcal vaccine development. Clin. Exp. Vaccine Res. 7, 76–81. 10.7774/cevr.2018.7.1.76, PMID: 29399583 PMC5795048

[ref49] Löfling J. Vimberg V. Battig P. Henriques-Normark B. (2011). Cellular interactions by LPxTG-anchored pneumococcal adhesins and their streptococcal homologues. Cell. Microbiol. 13, 186–197. 10.1111/j.1462-5822.2010.01560.x, PMID: 21199258

[ref50] Madrid L. Seale A. C. Kohli-Lynch M. Edmond K. M. Lawn J. E. Heath P. T.. (2017). Infant group B streptococcal disease incidence and serotypes worldwide: systematic review and meta-analyses. Clin. Infect. Dis. 65(Suppl. 2), S160–S172. 10.1093/cid/cix656, PMID: 29117326 PMC5850457

[ref51] Margarit I. Rinaudo C. Galeotti C. Maione D. Ghezzo C. Buttazzoni E.. (2009). Preventing bacterial infections with pilus-based vaccines: the group B streptococcus paradigm. J. Infect. Dis. 199, 108–115. 10.1086/595564, PMID: 19086816

[ref52] Megiddo I. Klein E. Laxminarayan R. (2018). Potential impact of introducing the pneumococcal conjugate vaccine into national immunisation programmes: An economic-epidemiological analysis using data from India. BMJ Glob. Health 3:636. 10.1136/bmjgh-2017-000636, PMID: 29765775 PMC5950640

[ref53] Mensah D. Asampong R. Amuna P. Ayanore M. A. (2020). COVID-19 effects on national health system response to a local epidemic: the case of cerebrospinal meningitis outbreak in Ghana. Pan Afr. Med. J. 35(Suppl. 2):14. 10.11604/pamj.2020.35.2.23138, PMID: 32528625 PMC7266470

[ref54] Michel J. L. Madoff L. C. Kling D. E. Kasper D. L. Ausubel F. M. (1991). Cloned alpha and beta C-protein antigens of group B streptococci elicit protective immunity. Infect. Immun. 59, 2023–2028. 10.1128/IAI.59.6.2023-2028.1991, PMID: 1674738 PMC257960

[ref55] Moriel D. Scarselli M. Serino L. Mora M. Rappuoli R. Masignani V. (2008). Genome-based vaccine development: a short cut for the future. Hum. Vaccin. 4, 184–188. 10.4161/hv.4.3.6313, PMID: 20686357

[ref56] Nagao P. E. (2015). *Streptococcus agalactiae* (group B streptococci). Mol. Med. Microbiol. 3, 1751–1767. 10.1016/B978-0-12-397169-2.00099-8

[ref57] Neto M. (2008). Group B streptococcal disease in Portuguese infants younger than 90 days. Arch. Dis. Child. Fetal Neonatal Ed. 93, 90–93. 10.1136/adc.2007.127464, PMID: 18089629

[ref58] Neto A. Lavado P. Flores P. Al E. (2003). Risk factors for the nasopharyngeal carriage of respiratory pathogens by Portuguese children: phenotype and antimicrobial susceptibility of Haemophilus influenzae and *Streptococcus pneumoniae*. Microb. Drug Resist. 9, 99–108. 10.1089/107662903764736409, PMID: 12705689

[ref900] Nielsen S. Henrichsen J. (1992). Capsular types of Streptococcus pneumoniae isolated from blood and CSF during 1982–1987. Clin. Infect. Dis. 15, 794–798. 10.1093/clind/15.5.794, PMID: 1445978

[ref59] Nocard N. Mollereau R. (1887). Sur une mammite contagieuse des vaches laitieres. Ann. Inst. Pasteur 1, 109–126.

[ref60] Nuccitelli A. Rinaudo C. Maione D. (2015). Group B streptococcus vaccine: state of the art. Ther. Adv. Vaccines 3, 76–90. 10.1177/2051013615579869, PMID: 26288735 PMC4530403

[ref73] Obaro S. Monteil M. Henderson D. (1996). Fortnightly review: the pneumococcal problem. BMJ 312:1521. 10.1136/bmj.312.7045.1521, PMID: 8646147 PMC2351282

[ref61] Palmeiro J. De Carvalho N. Botelho A. Fracalanzza S. Madeira H. Dalla-Costa L. (2011). Maternal group B streptococcal immunization: capsular polysaccharide (CPS)-based vaccines and their implications on prevention. Vaccine 29, 3729–3730. 10.1016/j.vaccine.2011.02.102, PMID: 21414381

[ref62] Persson E. Berg S. Bevanger L. Bergh K. Valsö-Lyng R. Trollfors B. (2008). Characterisation of invasive group B streptococci based on investigation of surface proteins and genes encoding surface proteins. Clin. Microbiol. Infect. 14, 66–73. 10.1111/j.1469-0691.2007.01877.x, PMID: 18034863

[ref63] Pichichero M. E. (2017). Pneumococcal whole-cell and protein-based vaccines: changing the paradigm. Expert Rev. Vaccines 16, 1181–1190. 10.1080/14760584.2017.1393335, PMID: 29130395 PMC6277969

[ref64] Robbins J. Austrian R. Lee C. Rastogi S. Schiffman G. Henrichsen J.. (1983). Considerations for formulating the second-generation pneumococcal capsular polysaccharide vaccine with emphasis on the cross-reactive types within groups. J. Infect. Dis. 148, 1136–1159. 10.1093/infdis/148.6.1136, PMID: 6361173

[ref65] Russell F. Carapetis J. Satzke C. Tikoduadua L. Waqatakirewa L. Chandra R. (2010). Pneumococcal nasopharyngeal carriage following reduced doses of a 7-valent pneumococcal conjugate vaccine and a 23-valent pneumococcal polysaccharide vaccine booster. Clin. Vaccine Immunol. 17, 1970–1976. 10.1128/CVI.00117-10, PMID: 20943882 PMC3008188

[ref66] Russell N. J. Seale A. C. O’Driscoll M. O’Sullivan C. Bianchi-Jassir F. Gonzalez-Guarin J.. (2017). Maternal colonization with group B Streptococcus and serotype distribution worldwide: systematic review and meta-analyses. Clin. Infect. Dis. 65(Suppl. 2), S100–S111. 10.1093/cid/cix658, PMID: 29117327 PMC5848259

[ref67] Salloum M. van der Mee-Marquet N. Valentin-Domelier A. Quentin R. (2011). Diversity of prophage DNA regions of *Streptococcus agalactiae* clonal lineages from adults and neonates with invasive infectious disease. PLoS One 6:e20256. 10.1371/journal.pone.0020256, PMID: 21633509 PMC3102099

[ref68] Sandgren A. Albiger B. Orihuela C. Tuomanen E. Normark S. Henriques-Normark B. (2005). Virulence in mice of pneumococcal clonal types with known invasive disease potential in man. J. Infect. Dis. 192, 791–800. 10.1086/432513, PMID: 16088828

[ref69] Sandgren A. Sjostrom K. Olsson-Liljequist B. Christensson B. Samuelsson A. Kronvall G.. (2004). Effect of clonal and serotype-specific properties on the invasive capacity of *Streptococcus pneumoniae*. J. Infect. Dis. 189, 785–796. 10.1086/381686, PMID: 14976594

[ref70] Santillan D. Rai K. Santillan M. Krishnamachari Y. Salem A. Hunter S. (2011). Efficacy of polymeric encapsulated C5a peptidase-based group B streptococcus vaccines in a murine model. Am. J. Obstet. Gynecol. 205, e1–e8. 10.1016/j.ajog.2011.06.024, PMID: 21802065 PMC3213321

[ref71] Schrag S. Zywicki S. Farley M. Reingold A. Harrison L. Lefkowitz L.. (2000). Group B streptococcal disease in the era of intrapartum antibiotic prophylaxis. N. Engl. J. Med. 342, 15–20. 10.1056/NEJM200001063420103, PMID: 10620644

[ref72] Seale A. C. Blencowe H. Bianchi-Jassir F. Embleton N. Bassat Q. Ordi J.. (2017). Stillbirth with group B Streptococcus disease worldwide: systematic review and meta-analyses. Clin. Infect. Dis. 65(Suppl. 2), S125–S132. 10.1093/cid/cix585, PMID: 29117322 PMC5850020

[ref74] Sinha A. Russell L. Tomczyk S. Verani J. Schrag S. Berkley J.. (2016). Disease burden of group B Streptococcus among infants in sub-Saharan Africa: a systematic literature review and meta-analysis. Pediatr. Infect. Dis. J. 35, 933–942. 10.1097/INF.0000000000001233, PMID: 27213263 PMC6858852

[ref75] Teatero S. Ferrieri P. Martin I. Demczuk W. McGeer A. Fit-tipaldi N. (2017). Serotype distribution, population structure, and antimicrobial resistance of group B streptococcus strains recovered from colonized pregnant women. J. Clin. Microbiol. 55, 412–422. 10.1128/JCM.01615-16, PMID: 27852675 PMC5277510

[ref76] Tettelin H. Nelson K. E. Paulsen I. T. Eisen J. A. Read T. D. Peterson S.. (2001). Complete genome sequence of a virulent isolate of *Streptococcus pneumoniae*. Science 293, 498–506. 10.1126/science.1061217, PMID: 11463916

[ref77] Thigpen M. Whitney C. Messonnier N. Zell E. Lynfield R. Hadler J.. (2011). Bacterial meningitis in the United States, 1998-2007. N. Engl. J. Med. 364, 2016–2025. 10.1056/NEJMoa1005384, PMID: 21612470

[ref78] Torné A. Dias J. Quinten C. Hruba F. Busana M. Lopalco P.. (2014). European enhanced surveillance of invasive pneumococcal disease in 2010: data from 26 European countries in the post-heptavalent conjugate vaccine era. Vaccine 32, 3644–3650. 10.1016/j.vaccine.2014.04.066, PMID: 24795228

[ref79] Turrentine M. Ramirez M. (2008). Recurrence of group B streptococci colonization in subsequent pregnancy. Obstet. Gynecol. 112, 259–264. 10.1097/AOG.0b013e31817f5cb9, PMID: 18669720

[ref80] Weisner A. Johnson A. Lamagni T. Arnold E. Warner M. Heath P.. (2004). Characterization of group B streptococci recovered from infants with invasive disease in England and Wales. Clin. Infect. Dis. 38, 1203–1208. 10.1086/382881, PMID: 15127328

[ref81] Welte T. Torres A. Nathwani D. (2012). Clinical and economic burden of community-acquired pneumonia among adults in Europe. Thorax 67, 71–79. 10.1136/thx.2009.129502, PMID: 20729232

[ref82] White B. (1938). The Biology of Pneumococcus. New York, NY: The Commonwealth Fund.

[ref83] WHO (2015). WHO Recommendations for Prevention and Treatment of Maternal Peripartum Infections. Geneva: Switzerland. 26598777

[ref85] Wright A. Morgan W. Colebrook L. Dodgson R. (1914). Observations on prophylactic inoculation against pneumococcus infections, and on the results which have been achieved by it. Lancet 1, 87–95.

